# Uso da Terapia Diurética em Pacientes com Insuficiência Cardíaca Descompensada e Lesão Renal Aguda. O Que Fazer nesse Dilema?

**DOI:** 10.36660/abc.20210238

**Published:** 2021-04-08

**Affiliations:** 1 Universidade Federal de São Paulo Escola Paulista de Medicina Seção Cardiovascular São PauloSP Brasil Seção Cardiovascular, Disciplina de Geriatria e Gerontologia, Escola Paulista de Medicina da Universidade Federal de São Paulo, São Paulo, SP – Brasil; 2 Hospital Israelita Albert Einstein São PauloSP Brasil Hospital Israelita Albert Einstein, São Paulo, SP – Brasil

**Keywords:** Insuficiência Cardíaca/complicações, Insuficiênca Renal Crônica/complicações, Mortalidade, Saúde Pública, Envelhecimento, Hospitalização, Diuréticos/uso terapêutico, Creatinina, Marcadores Biológicos, Lipocalinas, Impedância Elétrica

A insuficiência cardíaca (IC) é um grave problema de saúde pública devido a sua alta prevalência, morbidade e mortalidade,^1^ liderando entre as causas de hospitalização nos Estados Unidos,^2^ A prevalência da doença aumenta com a idade, tornando os pacientes idosos ainda mais suscetíveis às repercussões desta doença.^1^ Isso aumenta a importância do tratamento preciso da IC e suas complicações, entre elas a IC descompensada (ICd).

Em pacientes com ICd, que necessitam de terapia diurética, é comum observamos a presença de lesão renal aguda (LRA) concomitante. A grande dúvida na hora de realizar o tratamento com diuréticos nas situações em que a função renal está alterada é saber o motivo da disfunção: Trata-se de um paciente ainda congesto, necessitando de otimização da terapia diurética (síndrome cardiorrenal tipo I)? Ou trata-se de um paciente que a terapia diurética foi realizada de forma excessiva, causando hipovolemia, o que levou a uma baixa perfusão renal (LRA pré-renal) ou até a uma necrose tubular aguda?

Essa dúvida ganha muita importância na prática clínica por implicar em abordagens terapêuticas diametralmente opostas nas duas situações: uma delas intensificar a terapia diurética e a outra suspender os diuréticos, ou até mesmo iniciar hidratação venosa parcimoniosa. E o fato destes pacientes muitas vezes serem idosos, multicomorbidos, em contexto de infecção concomitante, torna a leitura do perfil hemodinâmico um grande desafio clínico. Dificilmente um médico que trabalhe em unidades de emergência ou de terapia intensiva nunca esteve diante deste dilema.

**Figura 1:**
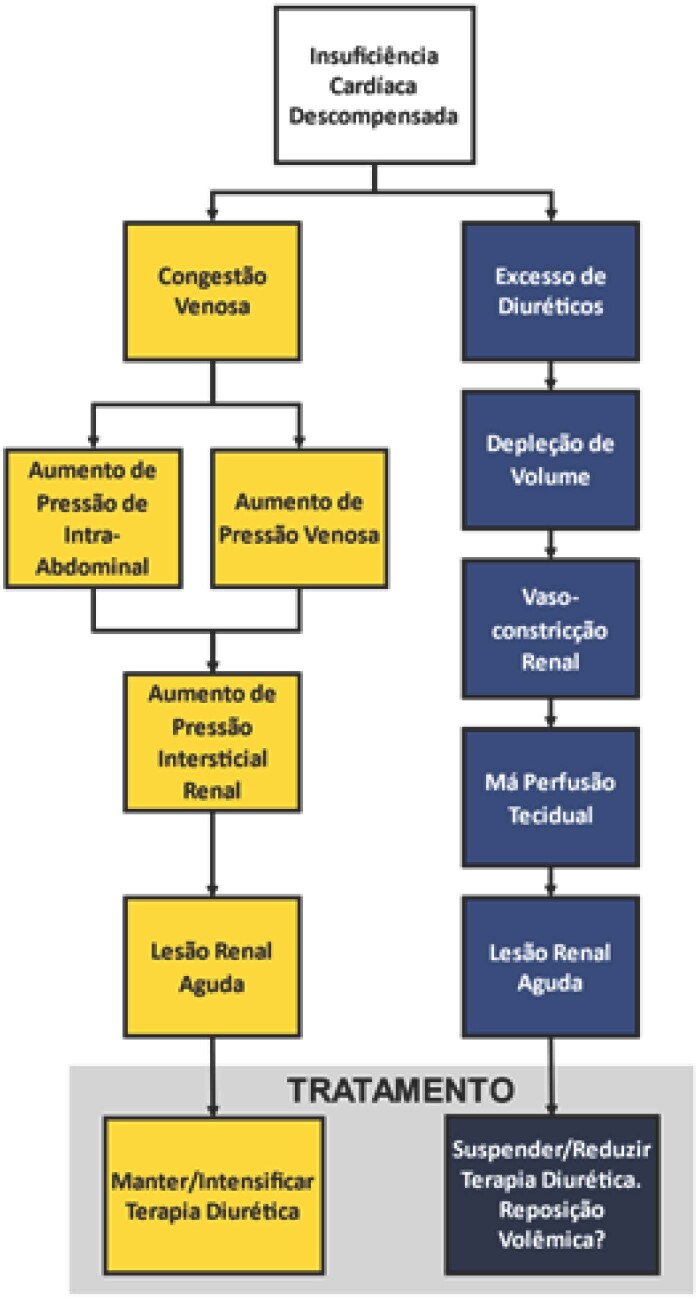
Representação esquemática da fisiopatologia e conduta em relação a terapia diurética de portadores de insuficiência cardíaca descompensada em tratamento.

Publicações anteriores corroboram esta dúvida, com alguns artigos entendendo a congestão como o grande fator associado a piora da lesão renal em pacientes com ICd, indicando uma terapia diurética mais agressiva,^3^^,^^4^ enquanto outros reconhecem o potencial efeito deletério da terapia diurética agressiva, entre eles a hipovolemia, indicando assim uma terapia diurética mais cautelosa,^5^ principalmente em pacientes idosos^6^ (Figura 1).

Os métodos laboratoriais e de imagem comumente disponíveis para acessar o perfil volêmico e hemodinâmico costumam pouco ajudar nesse quesito, já que não existe um método considerado padrão-ouro, tão pouco diretrizes ou protocolos que guiem quanto a melhor forma de responder essa questão. Métodos comumente usados em contexto de UTI, como a variação da pressão de pulso e a visualização da colapsibilidade das veias cavas superior e inferior, são validados apenas para a responsividade à infusão de fluídos, pouco ajudando quando a dúvida é a retirada de fluidos, além de serem eficazes apenas em pacientes em ventilação mecânica. Já a dosagem plasmática do peptídeo natriurético tipo B (BNP) e a do fragmento N-terminal do peptídeo natriurético tipo B (NT-ProBNP) tem uma importância bem estabelecida no diagnóstico e prognóstico da ICd, porém foi pouco estudada como uma ferramenta para acessar o perfil hemodinâmico nestes pacientes, com um estudo disponível mostrando uma performance ruim.^7^

O artigo de Villacorta et al.,^8^ reportado no atual volume dos Arquivos Brasileiros de Cardiologia,^8^ busca justamente investigar se o mecanismo da piora da função renal após o tratamento diurético agressivo em pacientes com ICd ocorre por congestão ou por lesão tubular renal. O artigo busca também avaliar se a presença de LRA durante o tratamento ou a presença de congestão no momento da alta atuam como preditores de desfecho após um episódio de ICd. Foram avaliados 85 pacientes, utilizando o NGAL como marcador para lesão tubular renal e o index de hidratação com a bioimpedância elétrica para definir a presença de congestão no momento da alta. O estudo chegou à conclusão de que a congestão persistente, mas não a LRA, está associada a piores desfechos em pacientes hospitalizados por ICd; além disso, mostrou que a LRA foi consequência da congestão, não de uma lesão tubular renal.

Os autores encerram o artigo^8^ mostrando algumas publicações que advogam a favor da terapia diurética agressiva e concluindo que, desde que a redução agressiva da congestão seja promovida, a LRA não terá impacto negativo nos desfechos.

O artigo^8^ acrescenta à visão atual sobre o assunto principalmente de duas maneiras. Primeiro, pelo simples fato de trazer para debate esse tema tão importante e comum na prática médica, mas relativamente pouco debatido e estudado. Segundo, por trazer algumas novidades na forma de avaliar a questão, mais precisamente o uso do NGAL, um marcador de lesão renal mais preciso e precoce que a creatinina, e o uso da bioimpedância elétrica para detectar congestão subclínica, o que aumentaria a precisão da avaliação e a capacidade de predizer desfechos.

Apesar disso, por se tratar de uma questão complexa e de difícil avaliação com os métodos disponíveis na prática médica, o manejo de pacientes com LRA em contexto de ICd ainda continua um enorme desafio clínico, com muitas perguntas e poucas respostas definitivas. Dessa forma, mais estudos são necessários para ampliar o entendimento sobre o assunto. No cenário atual, a individualização dos casos e a percepção clínica do avaliador ainda são fundamentais.
