# Supplementary material for: Uso da Terapia Diurética em Pacientes com Insuficiência Cardíaca Descompensada e Lesão Renal Aguda. O Que Fazer nesse Dilema?
Source: Arq Bras Cardiol. 2021 Apr 8;116(4):725–6. [Article in Portuguese] doi: 10.36660/abc.20210238 (PMC8121394; doi:10.36660/abc.20210238)
Supplement: Translation

# Translation: Use of Diuretic Therapy in Patients with Decompensated Heart Failure and Acute Kidney Injury. What to do in this Dilemma?

**Affiliations:** 1 São PauloSP Brazil Seção Cardiovascular, Disciplina de Geriatria e Gerontologia, Escola Paulista de Medicina da Universidade Federal de São Paulo, São Paulo, SP - Brazil; 2 São PauloSP Brazil Hospital Israelita Albert Einstein, São Paulo, SP - Brazil

**Keywords:** Heart Failure/complications, Renal Insufficiency Chronic/complications, Mortality, Health Public, Aging, Hospitalization, Diuretics, Creatinine, Markers Biological, Lipocalins, Electric Impedance

Heart failure (HF) is a severe public health problem due to its high prevalence, morbidity and mortality.^1^ It is the main cause of hospitalization in the United States.^2^ The prevalence of the disease increases with age, making elderly patients even more susceptible to the repercussions of this disease.^1^ This increases the importance of precise treatment of HF and its complications, including decompensated HF (dHF).

In patients with dHF, who require diuretic therapy, concomitant acute kidney injury (AKI) is a common finding. The big question when it comes to treatment with diuretics in situations where kidney function is altered is to know the reason for the dysfunction: Is the patient is still congested, requiring optimization of diuretic therapy (type I cardiorenal syndrome)? Or is it a patient whose diuretic therapy was carried out excessively, causing hypovolemia, which led to low renal perfusion (pre-renal AKI) or even an acute tubular necrosis?

This question gains a lot of importance in clinical practice because it implies diametrically opposed therapeutic approaches in both situations: intensifying diuretic therapy and discontinuing diuretics, or even initiating proper venous hydration. And the fact that these patients are often elderly, multicomorbid, in the context of concomitant infection, makes it clinically challenging to interpret the hemodynamic profile. It is hard to find an emergency or intensive care unit doctor that has never been faced with this dilemma.

Previous publications corroborate this issue, with some articles considering congestion as the major factor associated with worsening kidney injury in patients with dHF, indicating a more aggressive diuretic therapy,^3^^,^^4^ while others recognize the potentially harmful effect of aggressive diuretic therapy, including hypovolemia, thus indicating a more cautious diuretic therapy,^5^ especially in elderly patients^6^ (Figure 1).

**Figure 1:** Schematic representation of the physiopathology and management of diuretic therapy of patients with decompensated heart failure under treatment.

The laboratory and imaging methods commonly available to access the volume and hemodynamic profile are usually of little help in this regard, as there is no method considered to be the gold standard, nor are there any guidelines or protocols as to how best to answer this question. Methods commonly used in ICUs, such as variation in pulse pressure and assessment of collapsibility of superior and inferior vena cava, are validated only for responsiveness to the infusion of fluids and are of little help when it comes to fluid removal, and are only effective in mechanically ventilated patients. B-type natriuretic peptide (BNP) and N-terminal pro b-type natriuretic peptide (NT-ProBNP) plasma levels have a well-established importance in the diagnosis and prognosis of dHF. However, it has been little studied as a tool to access hemodynamic profile in these patients, with a study published showing poor performance.^7^

The article by Villacorta et al.,^8^ reported in the current volume of Arquivos Brasileiros de Cardiologia^8^, investigates whether the mechanism of worsening of renal function after aggressive diuretic treatment in patients with dHF occurs due to congestion or renal tubular injury. The article also assesses whether the presence of AKI during treatment or presence of congestion at discharge are predictors of outcome after an episode of dHF. Altogether, 85 patients were evaluated using NGAL as a marker for renal tubular injury and the hydration index with electrical bioimpedance to define the presence of congestion at discharge. It was found that persistent congestion, not AKI, is associated with worse outcomes in patients hospitalized for dHF; moreover, it showed that AKI was a consequence of congestion, rather than of a renal tubular injury.

The authors finish the article^8^ by showing some publications in favor of aggressive diuretic therapy and conclude that, as long as aggressive reduction of congestion is promoted, AKI will not have any adverse impact on the outcomes.

The article^8^ adds to the current view on the subject mainly in two ways. Firstly, for the simple fact that it discusses this very important and common theme in medical practice, but relatively little debated and studied. Secondly, for bringing some new ways of analyzing the issue, more precisely using NGAL, a marker of kidney injury that is faster and more accurate than creatinine, and the use of electrical bioimpedance to detect subclinical congestion, which would increase the accuracy of assessment and the ability to predict outcomes.

Despite this, because it is a complex issue that is hard to assess with the methods available in medical practice, management of patients with AKI in the context of dHF remains a huge clinical challenge, with many questions and few definitive answers. Therefore, further studies are needed to help understand the subject. In the current scenario, the individualization of cases and the clinical perception of the evaluator are still critical.
